# Can Sodium‐Glucose Co‐Transporter‐2 Inhibitors Improve Sleep Quality, Anxiety, and Quality of Life in Patients With Heart Failure?

**DOI:** 10.1002/clc.70190

**Published:** 2025-08-05

**Authors:** Ilke Erbay, Naile Eris Gudul, Ahmet Furkan Suner, Pelin Aladag, Umit Karacar, Ahmet Avci

**Affiliations:** ^1^ Department of Cardiology Bulent Ecevit University Faculty of Medicine Zonguldak Turkey; ^2^ Department of Public Health Çaycuma District Health Directorate Zonguldak Turkey

**Keywords:** anxiety, heart failure, quality of life, SGLT2 inhibitors, sleep quality

## Abstract

**Background:**

Sodium‐glucose co‐transporter‐2 (SGLT2) inhibitors improve cardiovascular outcomes in heart failure (HF), but their effect on sleep quality (SQ) and patient‐centered outcomes remains unclear.

**Objective:**

This study aims to evaluate the impact of SGLT2 inhibitor use on SQ, anxiety, and quality of life in patients with HF.

**Methods:**

This longitudinal observational study included 95 HF patients grouped by SGLT2 inhibitor use. A total of 79 patients (SGLT2 inhibitor group: 33; non‐SGLT2 inhibitor group: 46) completed a 6‐month follow‐up. Sleep quality was assessed using the Pittsburgh Sleep Quality Index (PSQI), anxiety with the Beck Anxiety Inventory (BAI), and quality of life with the Short Form‐36 (SF‐36). Subgroup analyses were conducted based on left ventricular ejection fraction (LVEF), and logistic regression was used to identify predictors of PSQI improvement.

**Results:**

At baseline, PSQI scores were slightly better in the SGLT2 inhibitor group (*p* = 0.036), while BAI and SF‐36 scores were similar. At follow‐up, the SGLT2 inhibitor group showed significant improvements in PSQI (*p* < 0.001) and BAI (*p* = 0.002), whereas no significant changes were observed in the non‐SGLT2 inhibitor group for either PSQI (*p* = 0.698) or BAI (*p* = 0.373). PSQI improvement was observed in SGLT2 users regardless of LVEF. In multivariate analysis, SGLT2 inhibitor use was an independent predictor of PSQI improvement (adjusted OR: 4.255; *p* = 0.010).

**Conclusion:**

SGLT2 inhibitor use was associated with improved SQ and reduced anxiety in patients with HF, suggesting symptom‐related benefits beyond cardiovascular effects.

## Graphical abstract

This study evaluated the impact of SGLT2 inhibitors on sleep quality, anxiety, and quality of life in patients with heart failure. A total of 95 patients were followed for 6 months and grouped based on SGLT2 inhibitor use. The SGLT2 inhibitor group showed a significantly higher rate of sleep quality improvement (65.2%, *p* < 0.001), accompanied by reductions in anxiety (BAI) and improvements across multiple SF‐36 domains. Logistic regression analysis confirmed SGLT2 inhibitor use as an independent predictor of sleep quality improvement (adjusted OR: 4.26, *p* = 0.010), with benefits observed regardless of LVEF status.

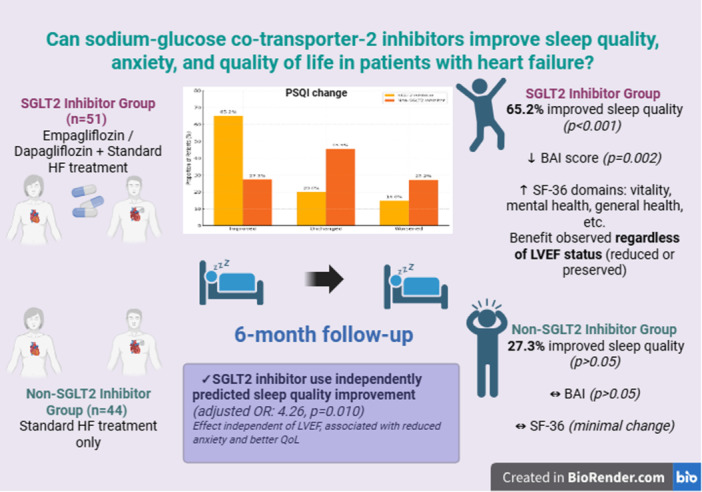

**Abbreviations**
BAI: Beck Anxiety Inventory
LVEF: left ventricular ejection fraction
OR: odds ratio
PSQI: Pittsburgh Sleep Quality Index
SF‐36: Short Form‐36 Health Survey
SGLT2: sodium‐glucose Co‐transporter 2

## 1 Introduction

Heart failure (HF) is an important public health problem affecting approximately 1%–2% of the adult population in developed countries [1, 2]. Despite advances in medical therapy, HF remains associated with significant morbidity, impaired quality of life (QoL), and high rates of hospitalization and mortality. Innovations in treatment strategies, especially the integration of new pharmacological agents into clinical practice, have led to remarkable changes in HF management. Among these agents, sodium‐glucose co‐transporter‐2 (SGLT2) inhibitors have emerged as a key treatment option not only to provide glycaemic control in diabetes mellitus (DM) but also to offer cardiovascular benefits in HF patients independent of the presence of diabetes status and left ventricular ejection fraction (LVEF), thereby reducing hospitalization rates and cardiovascular mortality [3, 4, 5].

There is substantial evidence that patients with HF experience significantly lower QoL compared with healthy individuals [6, 7, 8]. The assessment of QoL is a critical component of HF management, reflecting the extensive physical, mental and social burden associated with the disease. QoL indexes have become essential tools for assessing the efficacy and tolerability of different treatment modalities in HF [9]. Among these, the Short Form‐36 (SF‐36) health‐related QoL index is widely used and provides valuable insights for clinical decision making [9].

Sleep and anxiety disorders are frequently reported in patients with HF and are known to adversely affect clinical outcomes, treatment adherence, and overall well‐being [10, 11]. Improving QoL has become one of the central goals of modern HF management. Recent studies have demonstrated that SGLT2 inhibitors improve QoL in HF patients, primarily through gains in functional status and symptom relief [12, 13]. However, their effect on patient‐reported outcomes such as sleep quality (SQ) and anxiety remains unclear.

In this study, we aim to investigate the effects of SGLT2 inhibitors on SQ, anxiety, and health‐related quality of life in patients with heart failure.

## 2 Materials and Methods

### 2.1 Study Population

A total of 95 patients with HF, including those with reduced ejection fraction (LVEF ≤ 40%) and those with mildly reduced or preserved ejection fraction (LVEF > 40%), who consecutively presented to the cardiology outpatient clinic of Bülent Ecevit University Faculty of Medicine between January 2024 and September 2024, were included in this study. Of these, 51 patients received either empagliflozin or dapagliflozin (10 mg once daily) as part of their HF treatment, while 44 patients continued on standard therapy without an SGLT2 inhibitor.

Patients aged 18 years and older with a cardiologist‐confirmed diagnosis of HF determined according to guideline criteria at least 3 months before enrolment and receiving medical treatment in accordance with the guideline were included in the study [5, 14]. Patients were excluded if they were classified as New York Heart Association (NYHA) functional class IV, had type 1 DM, an estimated glomerular filtration rate (eGFR) < 25 mL/min/1.73 m², uncontrolled hypo‐ or hyperthyroidism, active or previously treated malignancy, diagnosed or treated major depressive disorder, obstructive sleep apnea or other known sleep disorders, any evidence of genitourinary disease, dementia or cognitive impairment, excessive alcohol consumption (>14 units/week), active substance abuse, or regular use of sedative, hypnotic, or antipsychotic medications. The study was approved by the ethics committee of Bülent Ecevit University (decision number: 2024/23).

### 2.2 Study Procedure

All patients were assessed at baseline and at 6‐month follow‐up using the Turkish validated versions of the Pittsburgh Sleep Quality Index (PSQI), the SF‐36 health survey, and the Beck Anxiety Inventory (BAI) to evaluate sleep quality, quality of life, and anxiety, respectively. All assessments were conducted by the same cardiology team, who were blinded to treatment allocation. Written informed consent was obtained from all participants before enrollment.

In addition to these patient‐reported outcomes, clinical parameters including B‐type natriuretic peptide (BNP) levels, eGFR, LVEF and NYHA functional class were assessed at both time points to evaluate changes in cardiac function and symptom status.

The primary objective was to assess 6‐month changes in clinical and patient‐reported outcomes within each group, according to SGLT2 inhibitor use.

### 2.3 Questionnaires

Nocturnal sleep quality was assessed using the PSQI. Higher scores on the PSQI indicate poorer sleep quality. The Turkish version of the PSQI was validated by Ağargün et al. [15]. The SF‐36 is a self‐assessment scale consisting of 36 items and eight sub‐dimensions. The physical component of the SF‐36 assesses physical function, physical role limitation, pain and general health perception, while the mental component assesses emotional role limitation, vitality, mental health and social functionality [16]. Lower scores indicate poorer quality of life. The Turkish validation of the SF‐36 was performed by Koçyiğit et al. [17]. Anxiety levels were assessed using the BAI, a 21‐item scale designed to measure the severity and frequency of anxiety symptoms. Higher scores indicate more severe anxiety. The Turkish version of the BAI was validated by Ulusoy et al. [18].

### 2.4 Follow‐Up

A total of 95 patients were initially enrolled in the study. During the 6‐month follow‐up period, cardiovascular death occurred in six patients, with four deaths in the non‐SGLT2 inhibitor group and 2 in the SGLT2 inhibitor group. Additionally, seven patients in the non‐SGLT2 inhibitor group were excluded from the analysis due to incomplete follow‐up; four patients withdrew consent and three were hospitalized for decompensated HF. In the SGLT2 inhibitor group, three patients were excluded from the analysis due to incomplete follow‐up; two withdrew consent and one was hospitalized for decompensation. After these exclusions, a total of 79 patients completed the follow‐up assessments and were included in the longitudinal (baseline‐to‐follow‐up) comparisons.

### 2.5 Statistical Analysis

All statistical analyses were performed using R software (version 4.4.3; R Foundation for Statistical Computing, Vienna, Austria). Continuous variables were presented as mean ± standard deviation (SD) or median with interquartile range (IQR), depending on the distribution assessed by the Shapiro‐Wilk test. Categorical variables were presented as frequencies and percentages. Comparisons between the two groups (SGLT2 inhibitor group and non‐SGLT2 inhibitor group) were performed using the Student's *t*‐test or Mann–Whitney *U* test for continuous variables, and the chi‐square test or Fisher's exact test for categorical variables, as appropriate. For within‐group comparisons of repeated measures (baseline vs. six‐month follow‐up), the paired *t*‐test or Wilcoxon signed‐rank test was used for continuous variables, depending on normality, and the McNemar's test for categorical variables. Group differences in PSQI improvement rates were compared using the chi‐square test. For subgroup analyses, patients were classified into four groups based on LVEF status and SGLT2 inhibitor use. Baseline and follow‐up values within each subgroup were compared using the Wilcoxon signed‐rank test. To identify independent predictors of improvement in sleep quality, univariate logistic regression analyses were first performed. Variables with *p* < 0.10 in the univariate analysis and those clinically relevant according to the literature (age, sex, BNP change, NYHA class change, and baseline LVEF) were included in the multivariate logistic regression model using a forward stepwise selection method. All statistical tests were two‐tailed and a *p*‐value of < 0.05 was considered statistically significant.

## 3 Results

### 3.1 Baseline Characteristics

Baseline characteristics of the 95 enrolled patients are summarized in Table 1. The SGLT2 inhibitor group had a significantly lower median age compared to the non‐SGLT2 inhibitor group. (median age: 65 [58–74] vs. 74 [64–78.7] years, *p* = 0.031). There were no significant differences between the groups in terms of body mass index (BMI, *p* = 0.548), LVEF (*p* = 0.349), NYHA functional class distribution (*p* = 0.863), or gender (*p* = 0.716). The prevalence of DM was higher in the SGLT2 inhibitor group (64.7% vs. 31.8%, *p* = 0.001). Use of beta‐blockers, mineralocorticoid receptor antagonists (MRAs), angiotensin‐converting enzyme (ACE) inhibitors or angiotensin receptor blockers (ARBs), angiotensin receptor–neprilysin inhibitors (ARNIs), and loop diuretics was comparable between groups (*p* > 0.05 for all). Baseline PSQI scores were significantly lower in the SGLT2 inhibitor group (5.0 [3.0–7.0] vs. 6.0 [4.2–8.0], *p* = 0.036), whereas BAI scores and all SF‐36 subscales showed no significant differences (*p* > 0.05, for all) (Table 2).

**Table 1:** Baseline demographic, clinical, laboratory, and echocardiographic characteristics of patients according to SGLT2 inhibitor use.

	Non‐SGLT2 inhibitor group (*n* = 44)	SGLT2 inhibitor group (*n* = 51)	*p* value
**Age, years**	74 (64.0–78.7)	65.0 (58.0–74.0)	0.031
**BMI (kg/m²)**	28.5 (25.2–31.1)	28.4 (26.1–31.1)	0.548
**Male, n(%)**	26 (59.1)	32 (62.7)	0.716
**DM, n(%)**	14 (31.8)	33 (64.7)	0.001
**HT, n(%)**	39 (88.6)	45 (88.2)	0.951
**CKD, n(%)**	8 (18.2)	9 (17.6)	0.946
**Etiology of cardiomyopathy**	0.378		
Nonischemic, n(%)	11 (25.0)	13 (25.5)	
Ischemic, n(%)	28 (63.6)	36 (70.6)	
Hypertrophic, n(%)	5 (11.4)	2 (3.9)	
**GFR (mL/min/1.73 m²)**	60.2 ± 28.1	69.2 ± 25.5	0.107
**Sodium (mmol/L)**	138.0 ± 3.7	138.7 ± 4.6	0.369
**Potassium (mmol/L)**	4.6 (4.1–4.8)	4.5 (4.1–4.8)	0.603
**Hemoglobin (g/dL)**	12.2 ± 2.1	12.4 ± 2.0	0.656
**BNP (pg/mL)**	323.0 (130.0–1348.7)	1037.0 (139.1–3722.0)	0.189
**Echocardiographic assessment**			
**LVEF (%)**	37.5 ± 10.5	39.3 ± 10.3	0.349
**LV Edd (mm)**			
	37.5 ± 10.5	39.3 ± 10.3	0.349
**LV Esd (mm)**	52.0 (46.2–56.7)	54.0 (48.0–58.0)	0.223
**LA diameter (mm)**			
	44.0 (39.5–49.5)	48.0 (40.2–52.7)	0.264
**SPAP (mmHg)**	43.1 ± 8.2	41.5 ± 6.2	0.737
**TAPSE (mm)**	29.0 (20.0–37.2)	25.0 (21.0–45.0)	0.748
**ECG rhythm**	0.262		
Sinus rhythm, n(%)	23 (52.3)	35 (68.6)	
AF, n(%)	18 (40.9)	14 (27.5)	
Pacemaker rhythm, n(%)	3 (6.8)	2 (3.9)	
**HR (bpm)**	78.0 (64.2–90.0)	76.0 (66.0–89.0)	0.917
**SBP (mmHg)**	127.3 ± 16.7	128.2 ± 17.5	0.819
**DBP (mmHg)**	77.5 (71.2–83.7)	77.0 (72.0–84.0)	0.973
**Smoking status**	0.515		
Never, n(%)	19 (43.2)	17 (33.3)	
Current smoker, n(%)	19 (43.2)		
	19 (43.2)		
Ex‐smoker, n(%)	6 (13.6)		
	6 (13.6)		
**Educational attainment**	—		
Less than high school, n(%)	35 (79.5)		
	36 (70.6)		
High school graduate, n(%)	9 (20.5)		
	12 (23.5)		
College degree or higher, n(%)	—		
	3 (5.9)		
**Orthopnea, n(%)**	19 (43.2)	25 (49.0)	0.569
**PND, n(%)**	19 (43.2)	25 (49.0)	0.569
**Medication**			
Furosemide, n(%)	26 (59.1)	30 (60.0)	0.929
Indapamide/HCT, n(%)	11 (25.0)	16 (31.4)	0.492
MRA, n(%)	23 (52.3)	34 (66.7)	0.153
ACE inhibitor/ARB, n(%)	26 (59.1)	38 (74.5)	0.110
ARNI, n(%)	—	3 (5.9)	—
Beta Blocker, n(%)	39 (88.6)	49 (96.1)	0.166
Empagliflozin, n(%)	—	16 (31.4)	—
Dapagliflozin, n(%)	—	35 (68.6)	—
**HF‐related hospitalization, n(%)**	3 (6.8)	1 (2.0)	0.812
**Mortality during follow‐up, n(%)**	4 (9.1)	2 (3.9)	0.411
**NYHA class**	0.863		
Class I	19 (43.2)	23 (45.1)	
Class II	16 (36.4)	16 (31.4)	
Class III	9 (20.4)	12 (23.5)	

Abbreviations: ACE, angiotensin‐converting enzyme; AF, atrial fibrillation; ARB, angiotensin receptor blocker; ARNI, angiotensin receptor‐neprilysin inhibitor; BMI, body mass index; BNP, B‐type natriuretic peptide; CKD, chronic kidney disease; DBP, diastolic blood pressure; DM, diabetes mellitus; ECG, electrocardiogram; Edd, end‐diastolic diameter; Esd, end‐systolic diameter; GFR, glomerular filtration rate; HCT, hydrochlorothiazide; HR, heart rate; HT, hypertension; LA, left atrium; LV, left ventricle; LVEF, left ventricular ejection fraction; MRA, mineralocorticoid receptor antagonist; NYHA, New York Heart Association; PND, paroxysmal nocturnal dyspnea; SBP, systolic blood pressure; SPAP, systolic pulmonary artery pressure; TAPSE, tricuspid annular plane systolic excursion.

**Table 2:** Baseline PSQI, BAI, and SF‐36 subscale scores sccording to SGLT2 inhibitor use.

	Non‐SGLT2 inhibitor group (*n* = 44)	SGLT2 inhibitor group (*n* = 51)	*p* value
**PSQI**	6.0 (4.2–8.0)	5.0 (3.0–7.0)	0.036
**BAI**	9.5 (6.0–17.7)	10.0 (5.0–18.0)	0.849
**Physical function**	56.4 ± 28.6	56.3 ± 30.4	0.943
**Physical role limitation**	0.0 (0.0–100)	0.0 (0.0–100.0)	0.693
**Emotional role limitation**	0.0 (0.0–100)	0.0 (0.0–100.0)	0.775
**Vitality**	49.7 ± 24.8	50.6 ± 20.5	0.787
**Mental health**	60.0 (40.0–80.0)	60.0 (40.0–80.0)	0.735
**Social functionality**	50.0 (37.5–75.0)	50.0 (25.0–100.0)	0.799
**Pain**	63.7 (32.5–100.0)	62.5 (42.5–90.0)	0.958
**General health perception**	46.5 ± 19.6	46.4 ± 18.9	0.922

Abbreviations: BAI, Beck Anxiety Inventory; BMI, body mass index; DM, diabetes mellitus; PSQI, pittsburgh sleep quality index; SF‐36, Short Form‐36 Health Survey; SGLT2, sodium‐glucose cotransporter‐2.

### 3.2 Six‐Month Follow‐Up Outcomes

Seventy‐nine patients completed the 6‐month follow‐up and were included in the longitudinal analysis. There were no significant changes in BMI, LVEF, left ventricular diameters, or systolic pulmonary artery pressure (SPAP) within either group during follow‐up (*p* > 0.05, for all). In the within‐group analysis, BNP levels significantly decreased after 6 months in both the SGLT2 inhibitor group (*p* < 0.001) and the non‐SGLT2 inhibitor group (*p* = 0.018) (Table 3).

**Table 3:** Changes in clinical, sleep, and quality‐of‐life parameters according to SGLT2 inhibitor use.

	Non‐SGLT2 inhibitor group (*n* = 33)			SGLT2 inhibitor group (*n* = 46)		
	Baseline	Follow‐up	*p* value	Baseline	Follow‐up	*p* value
**BMI (kg/m²)**	28.8 (25.4–31.3)	28.5 (25.2–31.1)	0.444	28.4 (26.1–31.1)	27.6 (25.1–29.5)	0.051
**LVEF (%)**	40.3 ± 10.3	39.2 ± 10.6	0.880	38.3 ± 10.3	39.1 ± 10.7	0.283
**BNP (pg/mL)**	323.0 (130.0–1348.7)	235.0 (79.0–500.0)	0.018	1037.0 (139.1–3722.0)	211.0 (99.5–894.9)	< 0.001
**LV Edd (mm)**	52.5 ± 7.7	54.3 ± 8.2	0.280	54.0 (48–58)	54.5 (49.7–59.1)	0.434
**LV Esd (mm)**	44.0 (39.5–49.5)	46.0 (40–49)	0.393	47.3 ± 9.7	46.1 ± 9.4	0.514
**SPAP (mmHg)**	30.4 ± 11.9	33.5 ± 11.1	0.891	36.5 ± 16.9	32.2 ± 13.9	0.541
**NYHA class** *						
** Class I (%)**	43.2	57.6	—	45.1	73.9	—
** Class II (%)**	36.4	24.2	—	31.4	21.8	—
** Class III (%)**	20.5	18.2	—	23.5	4.3	—
**Orthopnea (%)** *	43.2	18.2	—	49.0	15.2	—
**PND (%)** *	43.2	30.3	—	49.0	21.7	—
**PSQI score**	6.0 (4.2–8.0)	6.0 (4.0–8.0)	0.698	5.0 (3.0–7.0)	3.0 (3.0–6.0)	< 0.001
**BAI score**	9.5 (6.0–17.7)	10.0 (5.5–19.0)	0.922	10.0 (5.0–18.0)	8.0 (5.0–14.0)	0.002
**SF‐36**						
*** Physical function***	56.4 ± 28.6	61.6 ± 27.3	0.326	56.3 ± 30.4	61.4 ± 29.3	0.005
*** Physical role limitation***	0.0 (0.0–100.0)	50.0 (0.0–100.0)	0.042	0.0 (0.0–100.0)	50.0 (0.0–100.0)	0.004
*** Emotional role limitation***	0.0 (0.0–100.0)	33.0 (0.0–100.0)	0.244	66.0 (0.0–100.0)	66.0 (0.0–100.0)	0.026
*** Vitality***	49.7 ± 24.8	54.9 ± 25.9	0.090	50.6 ± 20.5	59.5 ± 20.6	< 0.001
*** Mental health***	60.0 (40.0–80.0)	60.0 (40.0–76.0)	0.761	60.0 (40.0–80.0)	60.0 (47.2–80.0)	0.010
*** Social functionality***	50.0 (37.5–75.0)	50.0 (50.0–75.0)	0.642	50.0 (25.0–100.0)	62.5 (34.4–100.0)	0.433
*** Pain***	63.7 (32.5–100)	67.5 (38.7–100)	0.285	62.5 (42.5–90)	67.5 (42.5–100)	0.249
*** General health perception***	46.5 ± 19.6	50.3 ± 20.7	0.028	46.4 ± 18.9	52.8 ± 20.3	< 0.001

Abbreviations: BAI, Beck Anxiety Inventory; BMI, body mass index; BNP, B‐type natriuretic peptide; Edd, end‐diastolic diameter; Esd, end‐systolic diameter; LVEF, left ventricular ejection fraction; NYHA, New York Heart Association; PND, paroxysmal nocturnal dyspnea; PSQI, Pittsburgh Sleep Quality Index; SF‐36, Short Form‐36 Health Survey; SGLT2, sodium‐glucose cotransporter‐2; SPAP, systolic pulmonary artery pressure.

* *p*‐values for categorical variables (e.g., NYHA class, orthopnea, PND) were not calculated due to missing follow‐up data in some patients (*n* = 79 at follow‐up).

NYHA functional class improved over time, with greater improvement observed in the SGLT2 inhibitor group. The proportion of patients in NYHA class I increased from 45.1% to 73.9%, while class III decreased from 23.5% to 4.3%. In the non‐SGLT2 inhibitor group, class I increased from 43.2% to 57.6% and class III decreased slightly from 20.5% to 18.2%. The prevalence of orthopnea and paroxysmal nocturnal dyspnea declined in both groups, with a greater reduction seen in the SGLT2 inhibitor group.

### 3.3 Patient Reported Outcomes

**Figure 1:**
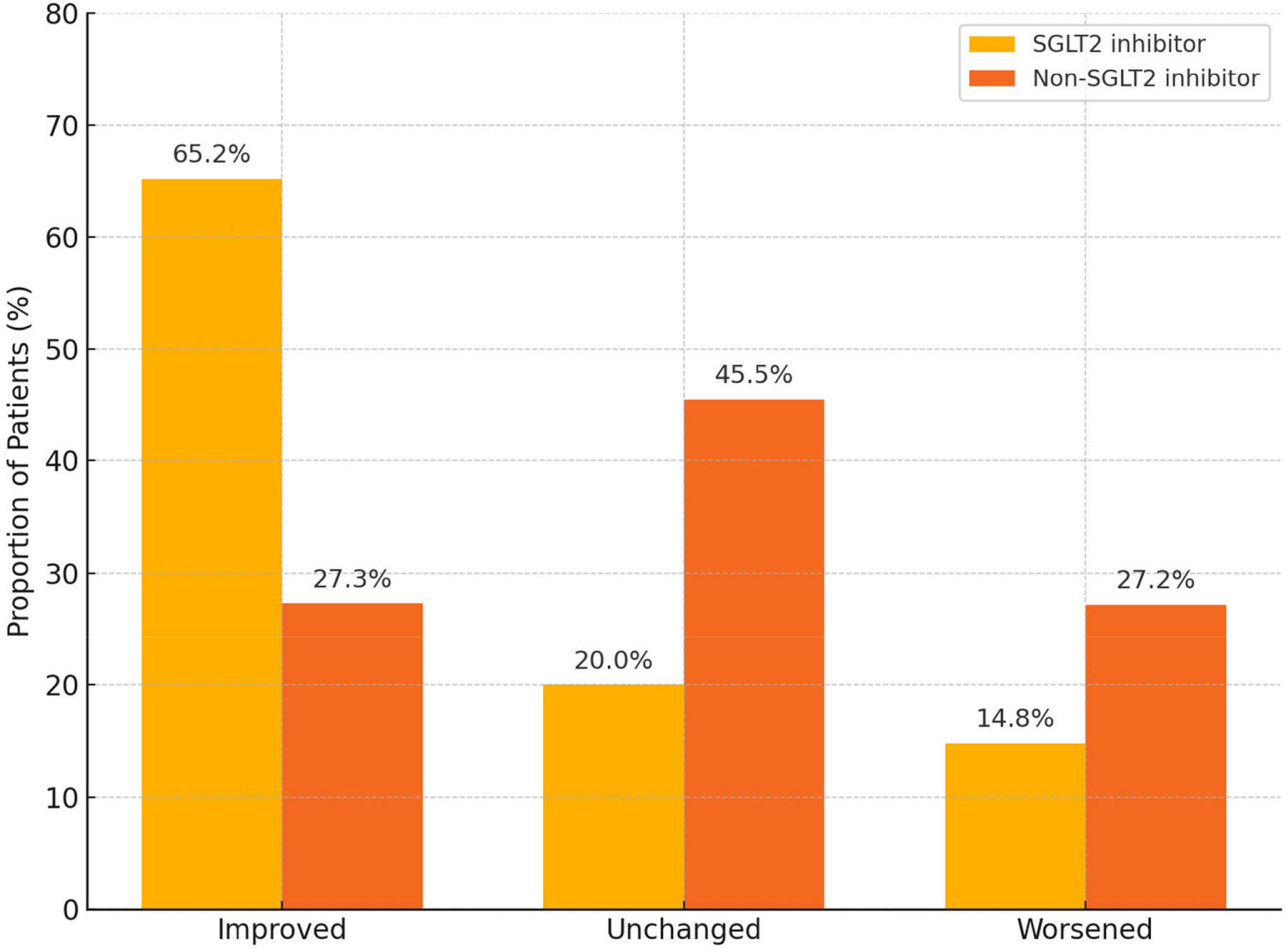
Proportion of patients by sleep quality change and SGLT2 inhibitor use. Improved sleep quality was defined as a reduction in PSQI score from baseline to 6‐month follow‐up. A greater proportion of patients in the SGLT2 inhibitor group showed improvement compared to the non‐SGLT2 inhibitor group (65.2% vs. 27.3%; *p* < 0.001). PSQI, Pittsburgh Sleep Quality Index; SGLT2, Sodium‐Glucose Co‐Transporter‐2.

During follow‐up, PSQI scores significantly decreased in the SGLT2 inhibitor group (*p* < 0.001), indicating improved subjective sleep quality, whereas no significant change was observed in the non‐SGLT2 inhibitor group (*p* = 0.698) (Table 3). Figure 1 shows the distribution of PSQI changes (improvement, unchanged, or worsening) between the two groups during the follow‐up.

In the SGLT2 inhibitor group, additional improvements were noted in several SF‐36 domains, including physical function (*p* = 0.005), physical role limitation (*p* = 0.004), emotional role limitation (*p* = 0.026), vitality (*p* < 0.001), mental health (*p* = 0.010), and general health perception (*p* < 0.001). Conversely, in the non‐SGLT2 inhibitor group, only physical role limitation (*p* = 0.042) and general health perception (*p* = 0.028) showed significant improvement, with no notable changes in other domains.

When the study population was stratified into four subgroups based on SGLT2 inhibitor use and EF status (reduced EF ≤ 40% and mildly reduced or preserved EF > 40%), subgroup analysis revealed further improvements within SGLT2 inhibitor users, particularly in those with reduced EF (Table 4). In patients with reduced EF receiving SGLT2 inhibitors (Group 1), significant improvements were observed in LVEF (*p* = 0.023), BNP (*p* < 0.001), PSQI (*p* = 0.004), BAI (*p* = 0.003) and several SF‐36 subscales. In patients with EF > 40% receiving SGLT2 inhibitors (Group 3), a significant improvement in PSQI was observed (*p* < 0.001). There were no statistically significant changes in any of the outcomes in patients not receiving SGLT2 inhibitors (Groups 2 and 4; all *p* > 0.05). Full SF‐36 subscale results are provided in Table S1.

**Table 4:** Changes in clinical and questionnaire outcomes by EF status and SGLT2 inhibitor use.

		Group 1 ( ↓ EF + SGLT2 + )	Group 2 ( ↓ EF + SGLT2–)	Group 3 ( ↑ EF + SGLT2 + )	Group 4 ( ↑ EF + SGLT2–)
**LVEF (%)**	Baseline	33.5 [25.5–37.0]	30.0 [25.8–37.0]	50.0 [45.0–52.0]	46.0 [45.0–50.0]
	Follow‐up	35.0 [25.0–40.0]	30.0 [27.5–40.0]	50.0 [45.0–53.0]	45.0 [40.5–50.0]
	*p*‐value	0.023	0.344	0.833	0.283
**BNP (pg/mL)**	Baseline	1248.0 [823.3–5360.8]	1138.0 [294.0–4259.8]	139.1 [76.0–749.0]	146.0 [102.3–426.0]
	Follow‐up	425.5 [156.0–1840.3]	250.0 [118.0–1830.0]	103.0 [37.5–274.0]	119.0 [67.0–244.0]
	*p*‐value	< 0.001	0.053	0.670	0.105
**PSQI**	Baseline	5.0 [3.0–7.0]	6.0 [4.3–8.0]	5.0 [3.0–6.0]	6.0 [3.5–7.0]
	Follow‐up	3.5 [2.3–6.0]	5.5 [4.0–8.0]	3.0 [3.0–5.0]	6.0 [4.3–7.8]
	*p*‐value	0.004	0.194	< 0.001	0.317
**BAI**	Baseline	12.0 [6.0–18.8]	12.0 [6.5–17.5]	8.0 [5.0–18.0]	8.5 [4.3–19.3]
	Follow‐up	8.0 [4.5–14.0]	12.0 [6.0–15.5]	8.0 [5.0–13.0]	10.0 [5.0–24.3]
	*p*‐value	0.003	0.422	0.247	0.242

*Note:* *Group definitions: Group 1 = LVEF ≤ 40% with SGLT2 inhibitor use, Group 2 = LVEF ≤ 40% without SGLT2 inhibitor use, Group 3 = LVEF > 40% with SGLT2 inhibitor use, Group 4 = LVEF > 40% without SGLT2 inhibitor use.

Only select clinical and quality‐of‐life outcomes are presented. Full SF‐36 subscales are available in Table S1.

Abbreviations: BAI, Beck Anxiety Inventory; BNP, B‐type natriuretic peptide; EF, ejection fraction; LVEF, left ventricular ejection fraction; PSQI, Pittsburgh Sleep Quality Index; SF‐36, Short Form‐36 Health Survey; SGLT2, sodium‐glucose cotransporter‐2.

### 3.4 Regression Analysis

In the multivariate logistic regression model, SGLT2 inhibitor use (adjusted odds ratio [aOR]: 4.255; 95% confidence interval [CI]: 1.415–12.796; *p* = 0.010), improvement in general health perception (aOR: 1.076; 95% CI: 1.004–1.154; *p* = 0.037), and reduction in anxiety scores (ΔBAI) (aOR: 0.803; 95% CI: 0.649–0.993; *p* = 0.043) were identified as independent predictors of sleep quality improvement (Table 5).

**Table 5:** Logistic regression analysis of predictors associated with PSQI improvement.

Variables	OR (95% CI)	*p* value	aOR (95% CI)**	*p* value
**Age**	1.004 (0.976–1.033)	0.797		
**Gender**	0.762 (0.332–1.746)	0.520		
**DM**	2.471 (1.081–5.674)	0.032		
**Baseline LVEF**	0.638 (0.238–1.438)	0.278		
**SGLT2 inhibitor use**	6.302 (2.580–15.394)	< 0.001	4.255 (1.415–12.796)	0.010
**Δ NYHA Class** *	1.580 (0.635–3.934)	0.326		
**Δ BNP** *	1.000 (1.000–1.000)	0.331		
**Δ BAI** *	0.735 (0.609–0.887)	0.001	0.803 (0.649–0.993)	0.043
**Δ SF‐36 Vitality**	1.083 (1.027–1.143)	0.003		
**Δ SF‐36 Mental health**	1.052 (1.002–1.104)	0.040		
**Δ SF‐36 Social function**	1.042 (1.002–1.083)	0.037		
**Δ SF‐36 General health perception**	1.102 (1.035–1.173)	0.002	1.076 (1.004–1.154)	0.037

Abbreviations: aOR, adjusted odds ratio; BAI, Beck Anxiety Inventory; BNP, B‐type natriuretic peptide; DM, diabetes mellitus; LVEF, left ventricular ejection fraction; NYHA, New York Heart Association; OR, odds ratio; PSQI, Pittsburgh Sleep Quality Index; SF‐36, Short Form‐36 Health Survey; SGLT2, sodium‐glucose cotransporter‐2.

*Δ indicates change from baseline to follow‐up.

** Variables with significant associations in univariate analysis (ΔBAI, ΔSF‐36 domains, SGLT2 inhibitor use, and DM) and clinically relevant covariates (age, gender, ΔBNP, ΔNYHA class, LVEF) were included in the multivariate logistic regression model. To avoid multicollinearity, only selected variables were retained in the final model.

## 4 Discussion

Our findings suggest that SGLT2 inhibitors may offer benefits beyond traditional cardiovascular endpoints, including meaningful improvements in SQ, anxiety and patient‐reported health status. These benefits were observed regardless of LVEF status and were more pronounced in patients receiving SGLT2 inhibitors. These findings suggest that SGLT2 inhibitors may offer benefits in areas such as sleep quality and quality of life, extending their clinical relevance beyond cardiometabolic control.

Quality of life is known to be significantly impaired in patients with HF, particularly in those with reduced LVEF. Previous studies have shown that improvements in functional capacity and the initiation of guideline‐directed medical therapy (GDMT) are associated with better QoL in this population [5, 19]. SGLT2 inhibitors have also been shown to improve QoL in landmark trials such as EMPEROR‐Reduced and DAPA‐HF, primarily through gains in functional status and symptom relief [12, 13]. More recently, a meta‐analysis by Gao et al. further confirmed these benefits across the spectrum of HF phenotypes [20]. However, the impact of SGLT2 inhibitors on more specific patient‐centered outcomes, such as sleep quality and anxiety, has not been adequately studied.

Sleep disturbances are highly prevalent among patients with HF and are strongly associated with poorer QoL, worse clinical outcomes, and increased symptom burden [21, 22, 23]. Longitudinal data from the Rotterdam Study also suggest that SQ progressively worsens in individuals with HF [24]. Redeker et al. reported that nearly 60% of HF patients suffer from poor SQ, which is closely linked to symptoms of anxiety and depression [10]. Furthermore, data from the Medical Outcomes Study showed that HF patients with sleep problems had significantly lower scores across all SF‐36 domains, emphasizing the multidimensional impact of impaired sleep [25]. Our findings align with this evidence, as improvements in SQ in the SGLT2 inhibitor group were paralleled by gains in several SF‐36 subscales.

Several mechanisms may explain the observed improvements in SQ, anxiety, and QoL with SGLT2 inhibitor therapy. These agents provide mild diuretic and natriuretic effects without triggering sympathetic nervous system activation [26, 27, 28]. This may help relieve symptoms of fluid overload such as orthopnea and paroxysmal nocturnal dyspnea, which are commonly associated with impaired sleep in patients with HF [29, 30]. In support of this, our study demonstrated a significant reduction in BNP levels after 6 months of SGLT2 inhibitors use, particularly in patients with reduced LVEF. This finding is consistent with previous large‐scale trials reporting that SGLT2 inhibitors lower natriuretic peptide levels in patients with HF with reduced EF [4, 28, 31]. In addition, we observed a favorable shift in NYHA functional class distribution and a marked decrease in the prevalence of orthopnea and paroxysmal nocturnal dyspnea among patients receiving SGLT2 inhibitors. While both groups showed some symptomatic relief, the magnitude of improvement was clearly greater among patients receiving SGLT2 inhibitors, suggesting a treatment‐specific effect. These improvements in objective clinical parameters suggest that relief from congestion and enhanced functional capacity may have played a role in better sleep, reduced anxiety, and a more positive perception of overall health. This observation was further supported by our subgroup analysis, which demonstrated consistent improvements in PSQI scores in both the reduced EF group and other EF groups. These findings align with the recent meta‐analysis by Shah and Turgeon [32], which demonstrated QoL benefits of SGLT2 inhibitors across the full spectrum of LVEF.

Supporting this interpretation, emerging evidence suggests that SGLT2 inhibitors may improve sleep‐related outcomes through multiple mechanisms. Armentaro et al. [33] reported improvements in AHI and nocturnal oxygen saturation in patients with HF treated with SGLT2 inhibitors. Neeland et al. [34] highlighted that a reduction in nocturnal rostral fluid shift may relieve upper airway congestion and contribute to better sleep quality. In our study, PSQI scores improved significantly without a corresponding change in BMI, suggesting that fluid redistribution rather than weight loss may have played a role. Notably, regression analysis identified SGLT2 inhibitor use as an independent predictor of SQ improvement, indicating a possible link between decongestion, improved functional capacity, and enhanced sleep.

This study has several limitations, including a relatively small sample size, especially in subgroup analyses, and a short follow‐up period (6 months), which may not reflect long‐term outcomes. The single‐center design may also limit external validity. Another limitation is the difference in baseline PSQI scores between groups, with the SGLT2 inhibitor group reporting slightly better SQ at baseline. However, the primary focus of our analysis was on changes within each group over time rather than direct between‐group comparisons. This design allowed us to evaluate treatment effects independently of baseline score differences. In addition, SQ was assessed using a subjective tool (PSQI) without objective sleep measurements such as polysomnography. Despite these limitations, the study is the first to evaluate the effect of SGLT2 inhibitors on both SQ and anxiety in patients with HF.

## 5 Conclusion

SGLT2 inhibitor therapy was associated with significant improvements in SQ, anxiety, and health‐related QoL in patients with HF, regardless of LVEF status. Future studies with larger populations and longer follow‐up will help to clarify these findings.

## Author Contributions

**Ilke Erbay:** study design, data collection, statistical analysis, data interpretation, manuscript preparation. **Naile Eris Gudul:** study design, data collection, data interpretation. **Ahmet Furkan Suner:** statistical analysis, manuscript preparation. **Pelin Aladag:** data interpretation, literature search. **Umit Karacar:** data collection. **Ahmet Avci:** manuscript preparation, literature search.

## Disclosure

The authors have nothing to report.

## Ethics Statement

The study was approved by the Ethics Committee of Bülent Ecevit University. The study was conducted in accordance with the principles of the Declaration of Helsinki.

## Consent

All authors consent to publication.

## Conflicts of Interest

The authors declare no conflicts of interest.

## Supporting information


**Supplementary Table 1:** Changes in SF‐36 subscale scores according to EF status and SGLT2 inhibitor use.

## Data Availability

The data that support the findings of this study are available on request from the corresponding author. The data are not publicly available due to privacy or ethical restrictions.
