# Supplementary material for: Can Sodium‐Glucose Co‐Transporter‐2 Inhibitors Improve Sleep Quality, Anxiety, and Quality of Life in Patients With Heart Failure?
Source: Clin Cardiol. 2025 Aug 5;48(8):e70190. doi: 10.1002/clc.70190 (PMC12322583; doi:10.1002/clc.70190)
Supplement: Supplementary file 1 — Supplementary Table 1: Changes in SF‐36 subscale scores according to EF status and SGLT2 inhibitor use. [file CLC-48-e70190-s001.docx]

**Supplementary Table 1.** Changes in SF-36 subscale scores according to EF status and SGLT2 inhibitor use

|  |  | **Group 1 (↓EF + SGLT2+)** | **Group 2 (↓EF + SGLT2–)** | **Group 3 (↑EF + SGLT2+)** | **Group 4 (↑EF + SGLT2–)** |
| --- | --- | --- | --- | --- | --- |
| **SF-36** | | | | | |
| ***Physical function*** | Baseline | 50.0 [30.0–80.0] | 60.0 [42.5–72.5] | 70.0 [30.0–95.0] | 62.5 [26.3–90.0] |
|  | Follow-up | 60.0 [37.5–80.0] | 65.0 [50.0–75.0] | 80.0 [55.0–95.0] | 67.5 [37.5–93.8] |
|  | *p*-value | 0.036 | 0.330 | 0.098 | 0.943 |
| ***Physical role*** | Baseline | 0.0 [0.0–87.5] | 0.0 [0.0–50.0] | 25.0 [0.0–100.0] | 17.5 [0.0–100.0] |
|  | Follow-up | 50.0 [0.0–100.0] | 50.0 [0.0–100.0] | 100.0 [12.5–100.0] | 75.0 [0.0–100.0] |
|  | *p*-value | 0.023 | 0.102 | 0.093 | 0.180 |
| ***Emotional role*** | Baseline | 0.0 [0.0–100.0] | 0.0 [0.0–100.0] | 0.0 [0.0–100.0] | 5.0 [0.0–100.0] |
|  | Follow-up | 33.0 [0.0–100.0] | 0.0 [0.0–100.0] | 100.0 [0.0–100.0] | 100.0 [0.0–100.0] |
|  | *p*-value | 0.066 | 0.581 | 0.180 | 0.180 |
| ***Vitality*** | Baseline | 47.5 [30.0–60.0] | 40.0 [26.3–60.0] | 55.0 [40.0–80.0] | 50.0 [31.3–80.0] |
|  | Follow-up | 50.0 [35.0–80.0] | 60.0 [32.5–77.5] | 65.0 [60.0–80.0] | 60.0 [31.0–83.8] |
|  | *p*-value | 0.001 | 0.106 | 0.007 | 0.715 |
| ***Mental health*** | Baseline | 56.0 [40.0–78.0] | 60.0 [40.0–71.5] | 60.0 [44.0–80.0] | 68.0 [40.0–80.0] |
|  | Follow-up | 60.0 [42.5–80.0] | 60.0 [40.0–76.0] | 60.0 [52.0–80.0] | 62.0 [40.5–78.0] |
|  | *p*-value | 0.035 | 0.878 | 0.119 | 0.475 |
| ***Social function*** | Baseline | 50.0 [25.0–84.4] | 50.0 [25.0–62.5] | 50.0 [50.0–100.0] | 60.0 [50.0–75.0] |
|  | Follow-up | 62.5 [25.0–93.8] | 50.0 [43.8–75.0] | 62.5 [50.0–100.0] | 68.8 [50.0–75.0] |
|  | *p*-value | 0.304 | 0.705 | 1.000 | 0.739 |
| ***Pain*** | Baseline | 51.3 [35.0–97.5] | 67.5 [32.5–100.0] | 67.5 [45.0–87.5] | 57.5 [25.0–97.5] |
|  | Follow-up | 67.5 [42.5–100.0] | 67.5 [38.8–100.0] | 67.5 [52.5–83.8] | 71.3 [28.1–100.0] |
|  | *p*-value | 0.202 | 0.655 | 1.000 | 0.317 |
| ***General health*** | Baseline | 50.0 [21.3–55.0] | 45.0 [25.0–50.0] | 50.0 [40.0–70.0] | 52.5 [30.0–65.0] |
|  | Follow-up | 50.0 [27.5–62.5] | 50.0 [25.0–60.0] | 60.0 [50.0–75.0] | 60.0 [32.5–65.0] |
|  | *p*-value | 0.012 | 0.080 | 0.007 | 0.196 |

*Group definitions: Group 1 = LVEF ≤40% with SGLT2 use, Group 2 = LVEF ≤40% without SGLT2 use, Group 3 = LVEF >40% with SGLT2 use, Group 4 = LVEF >40% without SGLT2 use.

**Abbreviations:** EF: Ejection fraction, LVEF: Left ventricular ejection fraction, SF-36: Short Form-36 Health Survey, SGLT2: Sodium-glucose cotransporter-2.
